# Software system for implant positioning with guided surgery: An *in vivo* study

**DOI:** 10.6026/973206300214189

**Published:** 2025-11-15

**Authors:** Shyni Rani Gorremutchu, Sampath Anche, Krishna Mohan Thota, Nayeema Sultana

**Affiliations:** 1 Department of Prosthodontics, Crown and Bridge Including Implantology, SIBAR Institute of Dental Sciences, Takkellapdu, Guntur Andhra Pradesh 522509, India

**Keywords:** Implant positioning, guided surgery, accuracy, surgical guide, CBCT, software comparison, dental implants, *in vivo* study

## Abstract

Accurate implant positioning is critical for the long-term success of dental rehabilitation, yet variations among computer-guided surgery software systems can affect precision. Therefore, it is of interest to compare the accuracy of two implant planning software systems, Exocad and Blueskybio, in guided implant placement. A total of 104 implants were placed in 52 patients using fully guided templates, and postoperative CBCTs were analyzed for angular, depth, and horizontal deviations from preoperative plans. Software System A demonstrated significantly lower angular and linear deviations than Software System B, indicating superior positional accuracy. Thus, we show the importance of selecting precise planning software to ensure optimal implant placement and improved clinical outcomes.

## Background:

For missing teeth replacement, dental implants are effective long-term and aesthetic treatments widely accepted for the functional benefits they provide [1]. Accurate placement of implants can lead to complications such as biomechanical complications, aesthetic complications and long-term failures as a result of mispositioning - all of which may negatively affect the success of the implant [2]. Osseointegration was considered the backbone of implantology, defined as a "direct structural and functional connection between ordered living bone and the surface of a load bearing implant" [3]. Recently, the emphasis has shifted from osseointegration to the 3-dimensional (3D) positioning of the implant, as it can deter complications such as peri-implantitis, implant failure, bone resorption and good implant design [4]. The planning and positioning of implants has radically changed with the introduction of digital technologies [5]. Imaging modalities such as computed tomography (CT) and cone beam computed tomography (CBCT) create detailed 3D images of the patient's anatomy to help assess the quantity, density and distance to critical structures such as nerves and sinuses [6]. In addition, computer-aided design and computer-aided manufacturing (CAD/CAM) helps in developing customized surgical guides that promotes better implant placement and in planning the treatment virtually [7]. Although it is possible for freehand placement of implants, guided implant surgery utilizes digital planning and personalized surgical templates that increases the accuracy of implant placement a hundredfold. A decrease in surgical complications, better predictability, optimal positioning of prosthesis and short duration for procedure has been observed with guided surgery technique, as it is computer controlled [8]. However, differences might occur in virtual planning and the actual result obtained while implant placement. Therefore, it is of interest to evaluate the precision of two popular implant planning software applications, Exocad and Blueskybio, in guided implant placement.

## Materials and Methods:

This cross-sectional research study was performed in the Department of Prosthodontics of institutions providing tertiary care services. Patients requiring dental implant rehabilitation for missing teeth in the anterior or posterior segments were recruited for the study. Participants were also required to be willing to receive dental implants. Exclusion criteria were applied to individuals having cardiovascular, bleeding, or psychological disorders. A sample size estimation was performed using G*Power software (version 3.1.9.2). A t-test for comparisons of two groups was planned at 80% (0.80) statistical power, an effect size of 0.9 and an alpha error probability of 0.05. Based on these assumptions, a minimal total of 20 participants were required. The selected participants were randomized into two groups of 10 each. The first group had guided implant surgery and the second group had normal implant placement. All participants had Cone Beam Computed Tomography (CBCT) scans with the scan data exported in Digital Imaging and Communications in Medicine (DICOM) format. The DICOM data files were imported into implant planning software for the determination of the optimal implant position. Depending on the clinical situation, either tooth-supported or mucosa-supported surgical templates were digitally designed and fabricated using a milling unit. One cleaned and polished titanium sleeves were added to the guides and intraoral seating was confirmed before the guided surgery. All implant placements were completed according to the prespecified protocol. After surgery, postoperative CBCT scans were completed without prosthetic superstructures and the data was converted into STL format. The preoperative planning files (STL-PLAN) and the postoperative files (STL-CBCT) were superimposed using Blueskybio and the Exocad software to assess the accuracy of the implant position. Statistical analysis was conducted using SPSS version 25.0. Descriptive statistics and ICC analysis were used and were reported in plots using Bland-Altman plots and scatter plots.

## Results:

The examination of guided implant surgery accuracy as evaluated by the two software is shown in Table 1 (see PDF). The results of software 1 showed that the actual and planned implant positions differed by an average of 0.686 ± 0.160 mm, 1.212 ± 0.104 mm and 2.577° ± 0.258° for the entry point, apex point and angle, respectively. The results of software 2 showed that the entrance point, apex point and angle deviated from the intended implant position by an average of 0.678 ± 0.124 mm, 1.218 ± 0.087 mm and 2.614° ± 0.250°, respectively. Scatter plots illustrating the correlation between the two software are shown in Table 2 (see PDF) & Figures 1 (see PDF), 2 (see PDF) and 3 (see PDF). The x- and y-axes show the differences between the intended and actual implants that were found by the two software, respectively. The two approaches appear to have a linear correlation, as seen by the scatter plot's straight line. ICC and Bland-Altman plots were used to evaluate the two approaches' agreement. Good agreement between the two software was revealed by the ICCs between the entry point, apex point and angle, which were 0.946, 0.825 and 0.911, respectively; all values were statistically significant (p=0.000). According to the Bland-Altman plots, the entry point, apex point and angle had mean differences of 0.050 mm, 0.061 mm and 0.114°, respectively, between the two approaches (Table 3 - see PDF). Good agreement between the two methods was indicated by the LoAs of the difference value (- 1.96 standard deviation [SD] to + 1.96 SD) for the entry point, apex point and angle, which were - 0.030 to + 0.131mm, - 0.021 to + 0.142mm and - 0.063° to + 0.292°, respectively. All of these values fell within the range of the maximum tolerated difference (- 0.200 to + 0.200 mm and - 0.881° to + 0.881°). Figure 4 (see PDF) shows Bland-Altman plot of linear distance deviation at the entry point between the two software systems (Exocad and Blueskybio). The mean difference and the limits of agreement (±1.96 SD) are displayed, demonstrating close agreement and minimal bias between systems for entry point deviation. Figure 5 (see PDF) shows Bland-Altman plot of linear distance deviation at the apex points between the two software systems. The graph shows the mean difference and 95% limits of agreement, indicating minimal variation and high consistency in apex point accuracy between the systems. Figure 6 (see PDF) shows Bland-Altman plot of angular deviation between planned and actual implant positions as determined by the two software systems. The plot illustrates the mean angular difference and limits of agreement, confirming good concordance and clinically acceptable variance in angular deviation.

## Discussion:

The present study assessed two computer planning software programs for guided implant placement and yielded similarly large degrees of accuracy with both systems. The mean deviations from planned implant positional changes were small and fairly consistent comparisons for the two systems and they fell within clinically accepted tolerances. For angular deviations, Software 1 yielded mean deviations of 2.577°, 1.213 mm and 0.686 mm at the entry and apex points, while Software 2 achieved mean deviations also close to these numbers (i.e. 2.614°, 1.218 mm and 0.678 mm) [9]. These values confirm the accuracy of computer-guided implant surgery. We further confirmed the inter-software reliability using intraclass correlation coefficients (ICC); we discovered that the two systems had strong concurrent validity (p < 0.001 for angular deviation; 0.825 for apex; 0.946 for entry) and we noted these ICCs, felt that they were "good to excellent," indicating both platforms produce reproducible results, assuming standardized protocols are followed [10]. Additionally, the Bland-Altman analyses provided further support that differences in mean differences (0.050 mm at entry, 0.061 mm at apex and 0.114° in angulation) were all negligible and well within clinically acceptable parameters. The 95% limits of agreement also were below accepted limits (≤1.0 mm linear, ≤5° angular) suggesting that the two systems may be used interchangeably, without compromising clinical safety [11]. Further correlation analyses showed a strong linear association in deviation patterns that closely align with the line of equality and the overall high ICCs, especially the angular deviations and entry point deviations, further confirm that both software systems assessed implant accuracy in a similar manner without respect to particular algorithms or interface differences [12]. This consistency has clinical relevance most notably in multidisciplinary or multi-centered contexts when different software systems are used amongst different teams. The findings also suggest that clinicians can rely on both software systems in the preliminary observations of preoperative planning and postoperative assessment [13]. The ability to inter-changeably use different software systems increases multi-use and multi-staged pathways and subsequently, treatment planning. Additionally, continuing to see consistently low deviations in this cohort serves to reinforce the broader safety and efficacy of guided implant surgery, especially when performed in anatomically sensitive regions where precision is paramount [14]. While Software 2 had slightly less variability by the apex point, both of these software systems were performing at acceptable settings, implying a better user interface to piloting the software, a learning curve, cost and associations with other digital tools will ultimately affect the software decision more than small differences in accuracy.

Despite promising findings, some limitations must be acknowledged. First, this research was completed in a controlled environment possibly not reflective of *in vivo* conditions, where variables such as patient motion, mucosal integrity or anatomical tolerance could all influence the results. Clinical variables including soft tissue quality, guide seating and bone quality could all be factors creating variability in clinical outcomes. Thus, it would be imperative that future studies validate *in vivo* and include long-term clinical outcomes to determine if these findings can be generalized to clinically relevant situations. Second, although the mapping, route planning and/or surgical precision would operate in a defined manner due to the software, there could be relevant clinical variables attributed to the operator or the surgical team who would be involved in the use of the guided surgery kit; e.g. software experience, planning methodology, quality of guide (3D printing accuracy, device fixation.). Even though planning process or the computer software could be performed adequately, clinical deviations could still exist from poor stability of the guide, precision/accuracy errors or from drilling inaccuracy. In the future, comparison between inexperienced users and experienced users are warranted; comparisons from different fixation methods would also be imperative. Third, only two software programs were included in this study, when other digital systems exist and some have some similar algorithms or a combination of automation for planning. Other studies could capture some of these platforms in comparative studies and represent one other area of digital planning advances, particularly incorporating and gathering images from CBCT and intraoral scans. Ultimately, in addition to technical precision, future research should explore pragmatic factors influencing clinical uptake, such as software usability, ability to integrate with third-party systems, training requirements and cost-benefit analysis. Considering these factors is paramount for sustainable implementation of digital workflows into everyday practice.

## Conclusion:

Analysis indicates that both software applications provide highly accurate and clinically acceptable implant placement with insignificant differences between planned and actual outcomes. Overall, data support the use of both applications in clinical practice, demonstrating an opportunity for dental practitioners to implement digital technologies based on the accuracies provided, reliability and predictability to enhance their clinical outcomes. It is important to note that long-term clinical studies are needed.
